# A novel two-way rebalancing strategy for identifying carbonylation sites

**DOI:** 10.1186/s12859-023-05551-2

**Published:** 2023-11-13

**Authors:** Linjun Chen, Xiao-Yuan Jing, Yaru Hao, Wei Liu, Xiaoke Zhu, Wei Han

**Affiliations:** 1 School of Computer Science, Wuhan University, Wuhan, China; 2 Guangdong Provincial Key Laboratory of Petrochemical Equipment Fault Diagnosis and School of Computer, Guangdong University of Petrochemical Technology, Maoming, China; 3 State Key Laboratory for Novel Software Technology, Nanjing University, Nanjing, China; 4 School of Computer and Information Engineering, Henan University, Kaifeng, China

**Keywords:** Protein carbonylation, Identifying protein carbonylation sites, Attention technique, Generative adversarial networks, Rebalance

## Abstract

**Background:**

As an irreversible post-translational modification, protein carbonylation is closely related to many diseases and aging. Protein carbonylation prediction for related patients is significant, which can help clinicians make appropriate therapeutic schemes. Because carbonylation sites can be used to indicate change or loss of protein function, integrating these protein carbonylation site data has been a promising method in prediction. Based on these protein carbonylation site data, some protein carbonylation prediction methods have been proposed. However, most data is highly class imbalanced, and the number of un-carbonylation sites greatly exceeds that of carbonylation sites. Unfortunately, existing methods have not addressed this issue adequately.

**Results:**

In this work, we propose a novel two-way rebalancing strategy based on the attention technique and generative adversarial network (Carsite_AGan) for identifying protein carbonylation sites. Specifically, Carsite_AGan proposes a novel undersampling method based on attention technology that allows sites with high importance value to be selected from un-carbonylation sites. The attention technique can obtain the value of each sample’s importance. In the meanwhile, Carsite_AGan designs a generative adversarial network-based oversampling method to generate high-feasibility carbonylation sites. The generative adversarial network can generate high-feasibility samples through its generator and discriminator. Finally, we use a classifier like a nonlinear support vector machine to identify protein carbonylation sites.

**Conclusions:**

Experimental results demonstrate that our approach significantly outperforms other resampling methods. Using our approach to resampling carbonylation data can significantly improve the effect of identifying protein carbonylation sites.

## Introduction

**Fig. 1:**
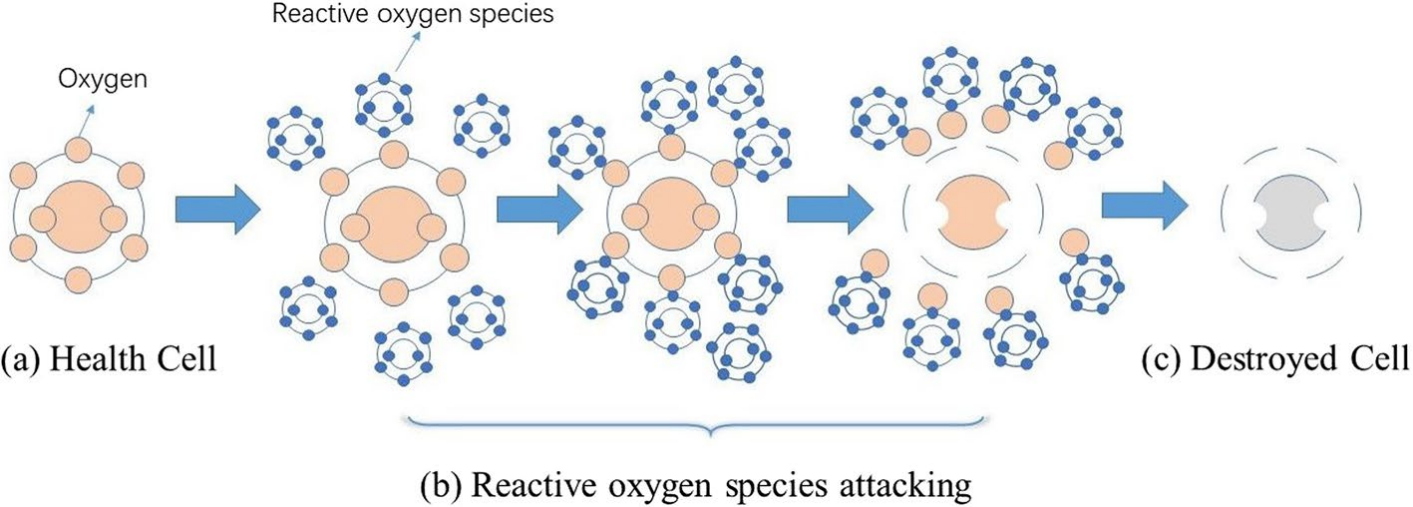
The process of protein carbonylation. **a** Health Cell. **b** Reactive oxygen species (ROS) attacking. Respectively, there are three subfigures that show ROS approaching the health cell, ROS attacking and capturing oxygen molecules in the health cell, and ROS leaving the destroyed cell. **c** Destroyed Cell

Protein carbonylation, that is after being attacked by reactive oxygen species, the side chains of amino acid residues are eventually converted into carbon-based products [1]. The process of carbonylation is shown in Fig. 1. From Fig. 1, we can see that carbonylation can change protein structure, and make protein loses its original biological function, eventually leading to disease. The level of protein carbonylation, as an indicator of protein oxidative damage, is used to evaluate the degree of the organism’s oxidation.

In addition, researchers have shown that protein carbonylation is involved in the etiology and pathophysiology of aging, apoptosis, and various neurodegenerative diseases. Carbonylation proteins cannot be repaired by the body’s antioxidant defense mechanisms, but slowly accumulate over time. When carbonylation proteins accumulate to a bad number, the functions of key enzymes in various signaling pathways are changed or even lost, which will lead to a series of diseases, such as aging, neurodegenerative diseases (such as Alzheimer’s disease, Parkinson’s disease, multiple Sexual sclerosis), inflammation, diabetes, tumors (such as uterine fibroids, malignant prostate cancer, breast cancer).

The level of protein carbonylation varies among different disease patients [1]. For example, glial fibrillary acidic protein (GFAP) has more carbonylation sites in multiple sclerosis [2], Pick’s disease [3], and aging patients, but few in Alzheimer’s disease patients [4]. In addition, B-actin has more carbonylation sites in Alzheimer’s disease and multiple sclerosis patients, but few in aging patients [2, 4].

According to the researchers, only four residues are particularly sensitive to carbonylation, and they are lysine (K), arginine (R), threonine (T), and proline (P) residues [5]. Carbonylation sites, used to indicate change or loss of carbonylation protein function, are crucial for understanding protein carbonylation processes and related complications [6]. The prediction jobs of carbonylation sites can enable clinicians to understand the occurrence probability and the corresponding number of carbonylation sites on the target protein, thus making appropriate therapeutic schemes [6].

### Motivation

In the past years, researchers have proposed a series of methods for predicting protein carbonylation sites [5, 7–13]. However, Carsite [14] found that there are imbalances in the carbonylation sites data, which can make the result of the classifier more biased towards the majority class. Therefore, CarSite used a one-sided selection (OSS) resampling method to balance the training dataset. It removes redundant samples and borderline samples by the condensed nearest-neighbor (CNN) method and Tomek Links.

CarSite-II [15] updated from CarSite, introduces and combines synthetic minority oversampling technique (SMOTE) [16] and K-means similarity-based undersampling (KSU) [17, 18] to construct balance training datasets. SMOTE was utilized to synthesize new simulation carbonylation sites (positive training samples) using true positive training samples. However, when the imbalance rate of the data set is very high, it will generate a lot of simulation samples and cause overfitting.

To solve the above problems, we propose a novel two-way rebalancing strategy based on attention technique and generative adversarial network (Carsite_AGan) for identifying protein carbonylation sites. Specifically, Carsite_AGan proposes a novel attention technique-based method to select sites with high importance value from un-carbonylation sites, to achieve un-carbonylation sites undersampling. The attention technique can obtain the value of each sample’s importance. In the meanwhile, Carsite_AGan designs a Gan-based method to generate high-feasibility positive carbonylation sites, to achieve carbonylation sites oversampling. The generative adversarial network (Gan) can generate high-feasibility samples through its generator and discriminator [19].

We propose a new resampling method rather than a new classifier for identifying carbonylation sites. Deep learning models have good performances in language recognition, automatic machine translation, photo translation, autonomous driving et al. [20–23], but adding it will make our model too complex. Nonlinear support vector machine [24] is a common classifier that is simpler than deep learning models. It is suitable not only for linear data but also for nonlinear data [24]. Therefore, Carsite_AGan uses a nonlinear SVM as the classifier for identifying protein carbonylation sites.

### Contribution

In this work, we propose a novel protein carbonylation sites prediction approach based on attention technique and generative adversarial network. The main contributions of our approach are as follows:

1. To achieve un-carbonylation sites undersampling, we propose a novel attention technique-based method to select sites with high importance value from un-carbonylation sites. The attention technique can obtain the value of each sample’s importance.
2. To achieve carbonylation sites oversampling, we design a Gan-based method to generate carbonylation sites with high feasibility. The generative adversarial network (Gan) can generate high-feasibility samples through its generator and discriminator.
3. Experimental results demonstrate that our approach significantly outperforms other resampling methods. Using our approach to rebalanced carbonylation data can significantly improve the effect of identifying protein carbonylation sites.

## Proposed method

**Fig. 2:**
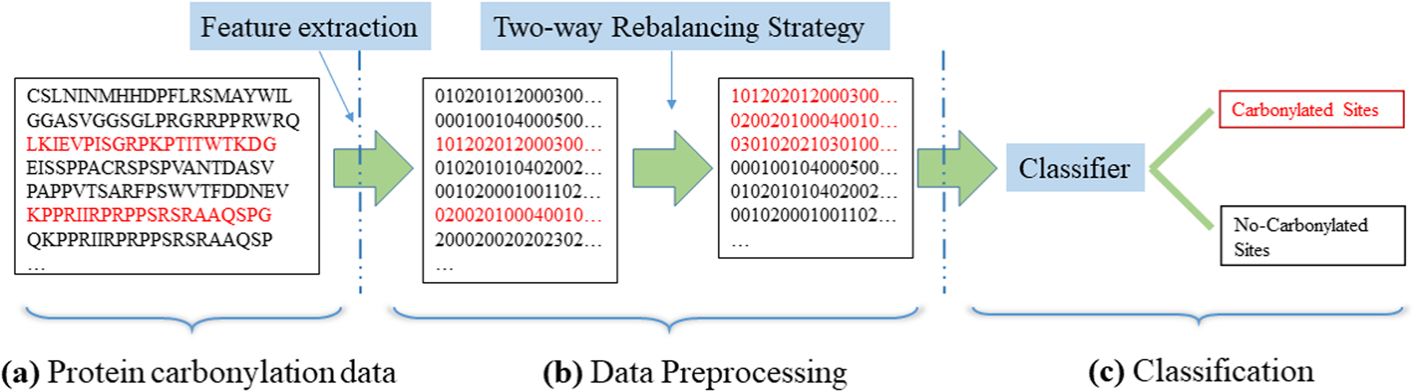
The flowchart of our approach. **a** We collect the protein carbonylation data. **b** In the data preprocessing stage, we use a features extraction strategy to convert protein carbonylation and non-carbonylation sequences into valid numerical vectors. Then Carsite_AGan normalizes the data and balances the categories. **c** Finally, we employ a classifier to classify the preprocessed data

In this work, we propose a novel two-way rebalancing strategy based on attention technique and generative adversarial network for identifying protein carbonylation sites. Figure 2 shows the flowchart of our approach. It contains three stages: the feature extraction, the two-way rebalancing strategy and the nonlinear support vector machine-based classification. Key notations used in this work are listed in Table 1.

**Table 1:** Summary of the key notations used in the paper

Notations	Explanations
$X_{neg} \in \mathbb {R} ^{d \times M}$	The negative samples set.
$X_{neg_i} \in \mathbb {R} ^d$	The i-th sample of negative samples set.
$Att1_i \in \mathbb {R} ^d$	The attention values between $X_{neg_i}$ and each sample of negative samples set.
*t*	The noise variable.
*pt*(*t*)	The prior of *t*.
$G(t,\theta )$	The generator that maps samples from the original space to the low-dimension space
	and consists of multi-layer perceptron.
$D(t,\theta )$	The discriminator and distribution of that represents the probability that sample
	comes from the true samples set instead of the generator.
$X_i \in \mathbb {R} ^d$	The i-th sample of samples set.
$y_i$	The i-th label of samples set.

### Feature extraction

We employ the distance-based residue features extraction strategy (DR) [25] to convert protein carbonylation and non-carbonylation sequences into valid numerical vectors. Given a protein sequence *Q* with *M* amino acid residues, i.e.

1 $$\begin{aligned} Q=Q_1Q_2...Q_i...Q_{M-1}Q_M \end{aligned}$$

where $Q_i$ represents the *i*th position amino acid residue along a given protein sequence. The sequence length is 21. The DR measure of *Q* can be defined as:

2 $$\begin{aligned} F_{d_{MAX}}\left( Q \right) =\left[ D_0\left( Q \right) ,D_1\left( Q \right) ,...,D_k\left( Q \right) ,...,D_{d_{MAX}-1}\left( Q \right) ,D_{d_{MAX}}\left( Q \right) \right] \end{aligned}$$

The dimension of $F_{d_{MAX}}\left( Q \right)$ is N + N x N x $d_{MAX}$, where N indicates N kinds of amino acid residues.

3 $$\begin{aligned} D_k\left( Q \right) ={\left\{ \begin{array}{ll} \left[ T_{A}^{0}\left( Q \right) ,T_{C}^{0}\left( Q \right) ,...,T_{Y}^{0}\left( Q \right) \right] \left( k=0 \right) \\ \left[ T_{AA}^{k}\left( Q \right) ,T_{AC}^{k}\left( Q \right) ,...,T_{YY}^{k}\left( Q \right) \right] \left( 1\leqslant k\leqslant d_{MAX} \right) \\ \end{array}\right. } \end{aligned}$$

where $i\in \left\{ A,C,D,E,F,G,H,I,K,L,M,N,P,Q,R,S,T,V,W,Y \right\}$, $T_{i}^{0}\left( Q \right)$ is the occurrences of the amino acid residue *i*, and $T_{ij}^{d}\left( Q \right)$ is the occurrences of the amino acid residue pair (*i*, *j*). $d_{MAX}$ represents the maximum distance between amino acid residue pair (*i*, *j*).

**Fig. 3:**
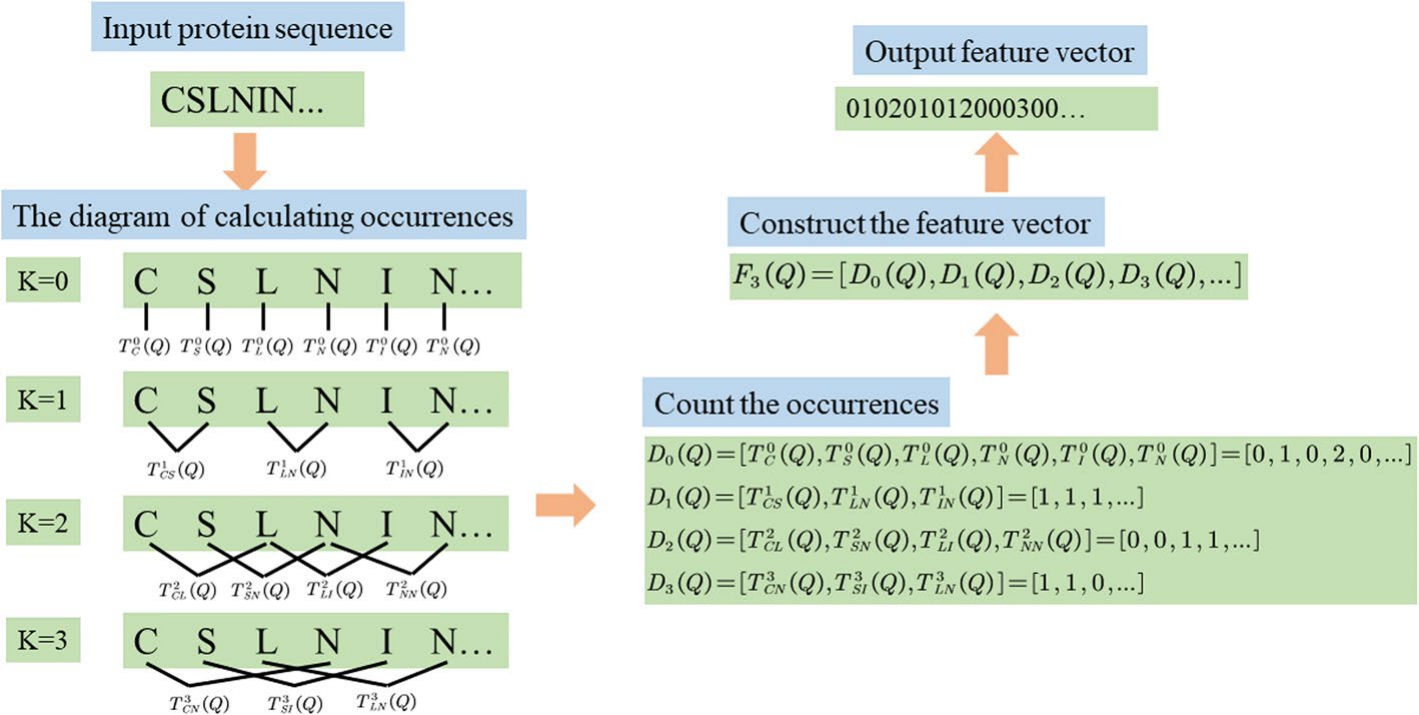
The process of generating DR feature vectors

Figure 3 shows the concrete process of generating DR feature vectors. In Fig. 3, researchers could further understand the concrete process of converting a carbonylation or no-carbonylation protein sequence into a valid numerical vector.

### Two-way rebalancing strategy

To achieve un-carbonylation sites undersampling, we propose a novel attention technique-based strategy to select sites with high importance value from un-carbonylation sites. The attention technique can obtain the value of each sample’s importance. Suppose there are M negative samples (un-carbonylation sites) and m positive samples (carbonylation sites), we can first obtain the optimal sampling scale s (the details of it can be found in the discussion section). Then we obtain the attention values of each negative sample and normalize them:

4 $$\begin{aligned} Att1_i\,\,=\,\,normalize\left( consine\left( X_{neg_i}, X_{neg} \right) \right) , \end{aligned}$$

where $Att1_{\left( i,i \right) }\in \left[ 0,1 \right]$ indicates the similarity between $X_{neg_i}$ and itself after normalization. When the value of $Att1_{\left( i,i \right) }$ is high, the similarity between $X_{neg_i}$ and itself after normalization is high, but the important value of $X_{neg_i}$ in the negative samples is low, otherwise high. Therefore, we select the negative samples corresponding to the bottom $\frac{s+M}{2}$ of $Att1_{\left( i,i \right) }$, to ensure that the selected negative samples have higher importance than others in the negative samples.

We compose the selected negative samples and positive samples into new samples $X'$. Then we obtain the attention values of each negative samples and normalize them:

5 $$\begin{aligned} Att2_i\,\,=\,\,normalize\left( consine\left( X'_{neg_i}, X' \right) \right) . \end{aligned}$$

We select the negative samples corresponding to the top *s* of $Att2_{(i, i)}$, to ensure that the selected negative samples have lower similarity with positive samples than others. In other words, the *s* negative samples we selected have high importance in the negative samples, but low similarity with positive samples. We achieve negative samples undersampling.

In the meanwhile, to achieve carbonylation sites oversampling, we use the Gan [19] to generate high-feasibility positive samples. The Gan is composed of a generator and a discriminator. The purpose of the generator $G(t; \theta )$ is to map the random input Gaussian noise into a fake sample. The purpose of discriminator $D(t; \theta )$ is to judge whether the input sample is fake. We train the generator $G(t; \theta )$ to minimize $log(1- D(G(t)))$. At the same time, We train the discriminator $D(t; \theta )$ to assign the correct label to true samples and simulation samples from the generator. We use the loss function of the Gan as follows:

6 $$\begin{aligned} \underset{G}{\min }\underset{D}{\max }V\left( D,G \right) =\mathbb {E} _{x~p_{data}\left( x \right) }\left[ \log D\left( x \right) \right] +\mathbb {E} _{t~p_t\left( t \right) }\left[ \log \left( 1-D\left( G\left( t \right) \right) \right) \right] , \end{aligned}$$

**Fig. 4:**
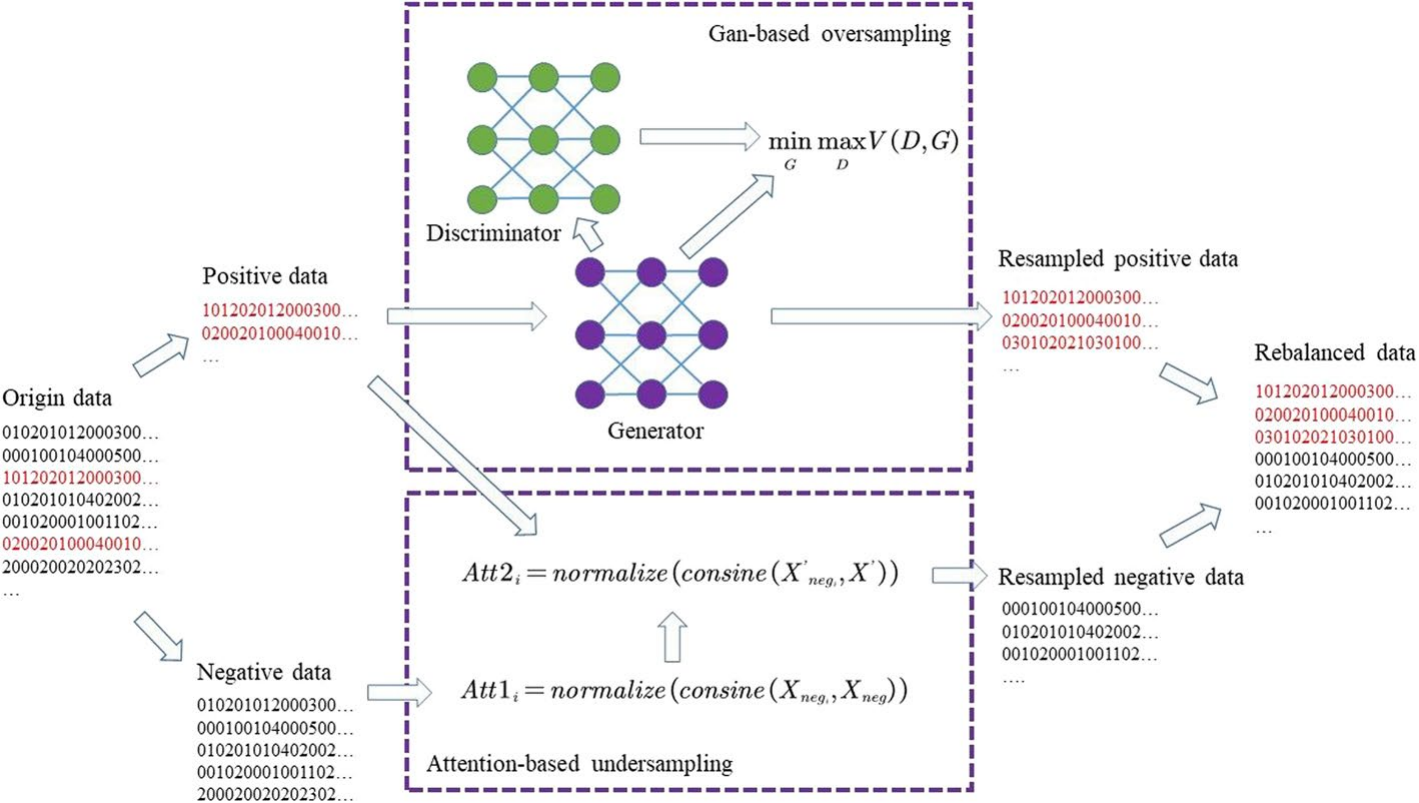
The details of the two-way rebalancing strategy

Finally, we use the trained generator $G(t; \theta )$ to generate high-feasibility samples, and achieve positive samples oversampling. We can see the details of the two-way rebalancing strategy in Fig. 4.

### Nonlinear support vector machine-based classification

To learn more information from rebalanced samples, we use a nonlinear support vector machine (SVM) as the classifier. Specially, we use the Gaussian kernel function to learn nonlinear information of resampling data. The Gaussian kernel function is as follows:

7 $$\begin{aligned} K\left( x,z \right) =\exp \left( -\frac{\left\| x-z \right\| ^2}{2\sigma ^2} \right) , \end{aligned}$$

Suppose training set is $T=\left\{ \left( x_1,y_1 \right) ,\left( x_2,y_2 \right) ,...,\left( x_N,y_N \right) \right\}$. We first employ the Karush-Kuhn-Tucker (KKT) conditions and construct the objective function as follows:

8 $$\mathop {\min }\limits_{\alpha } \frac{1}{2}\sum\limits_{{i = 1}}^{N} {\sum\limits_{{j = 1}}^{N} {\alpha _{i} \alpha _{j} y_{i} y_{j} \left( {x_{i} \cdot x_{j} } \right) - \sum\limits_{{i = 1}}^{N} {\alpha _{i} } } } s.t.\sum\limits_{{i = 1}}^{N} {\alpha _{i} y_{i} = 0} ,0 \le \alpha _{i} \le C,i = 1,2,...,N,$$

where *C* denotes the penalty parameter, the higher value of *C*, the heavy penalty for classification. The $\alpha$ denotes the KKT multiplier. Then we solve the objective function to obtain the optimal solution $\alpha ^*=\left( \alpha _{1}^{*},\alpha _{2}^{*},...,\alpha _{N}^{*} \right) ^T$. We obtain the bias $b ^*$ through the optimal solution $\alpha ^*$ as follows:

9 $$\begin{aligned} b^*=y_j-\sum _{i=1}^N{\alpha _{i}^{*}y_iK\left( x_i,x_j \right) }, \end{aligned}$$

Finally, we obatin the classification function as follows:

10 $$\begin{aligned} f\left( x \right) =sign\left( \sum _{i=1}^N{\alpha _{i}^{*}y_i\exp \left( -\frac{\left\| x-z \right\| ^2}{2\sigma ^2} \right) +b^*} \right) . \end{aligned}$$

## Experiments

**Fig. 5:**
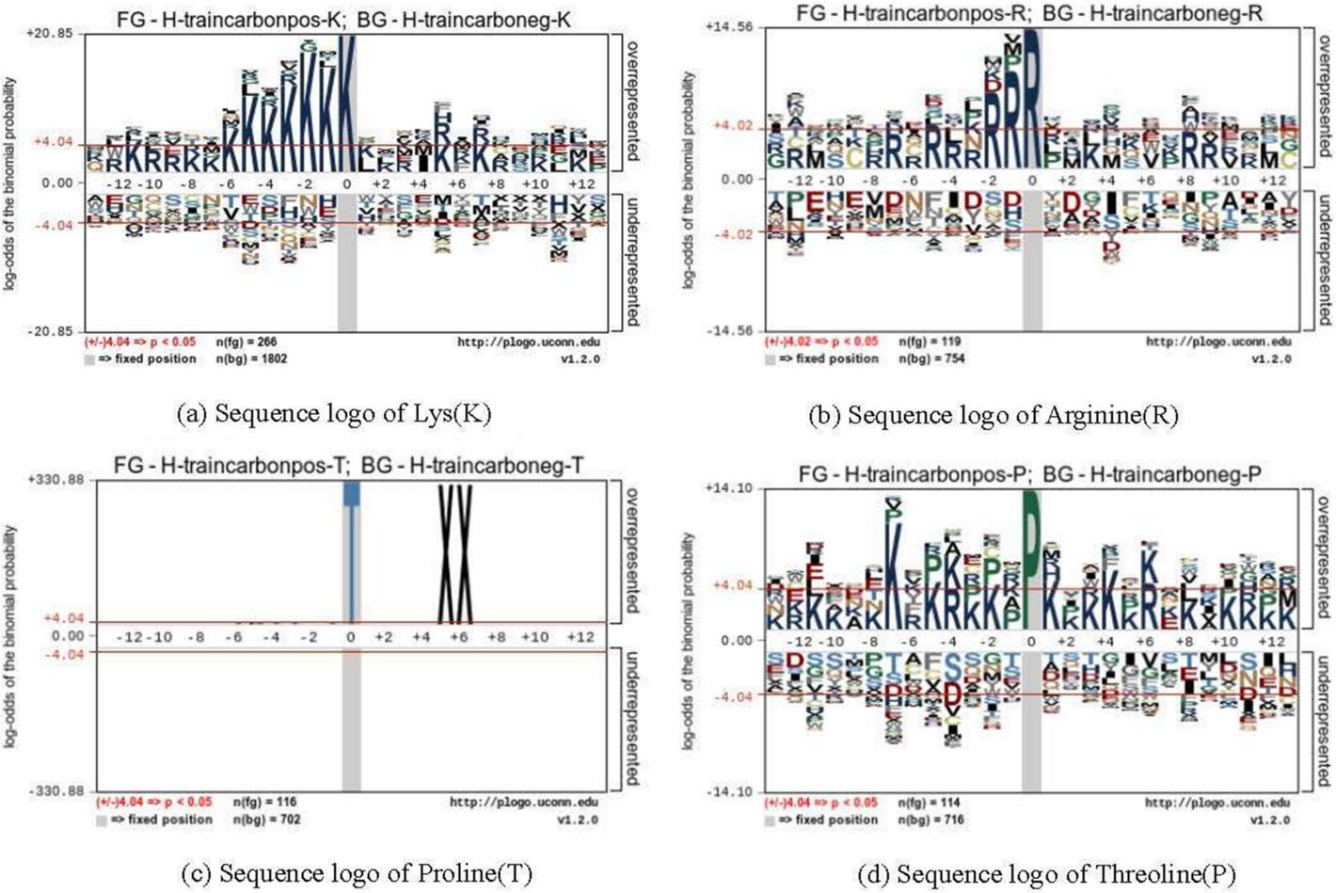
Sequence logo of four residues in training dataset

**Fig. 6:**
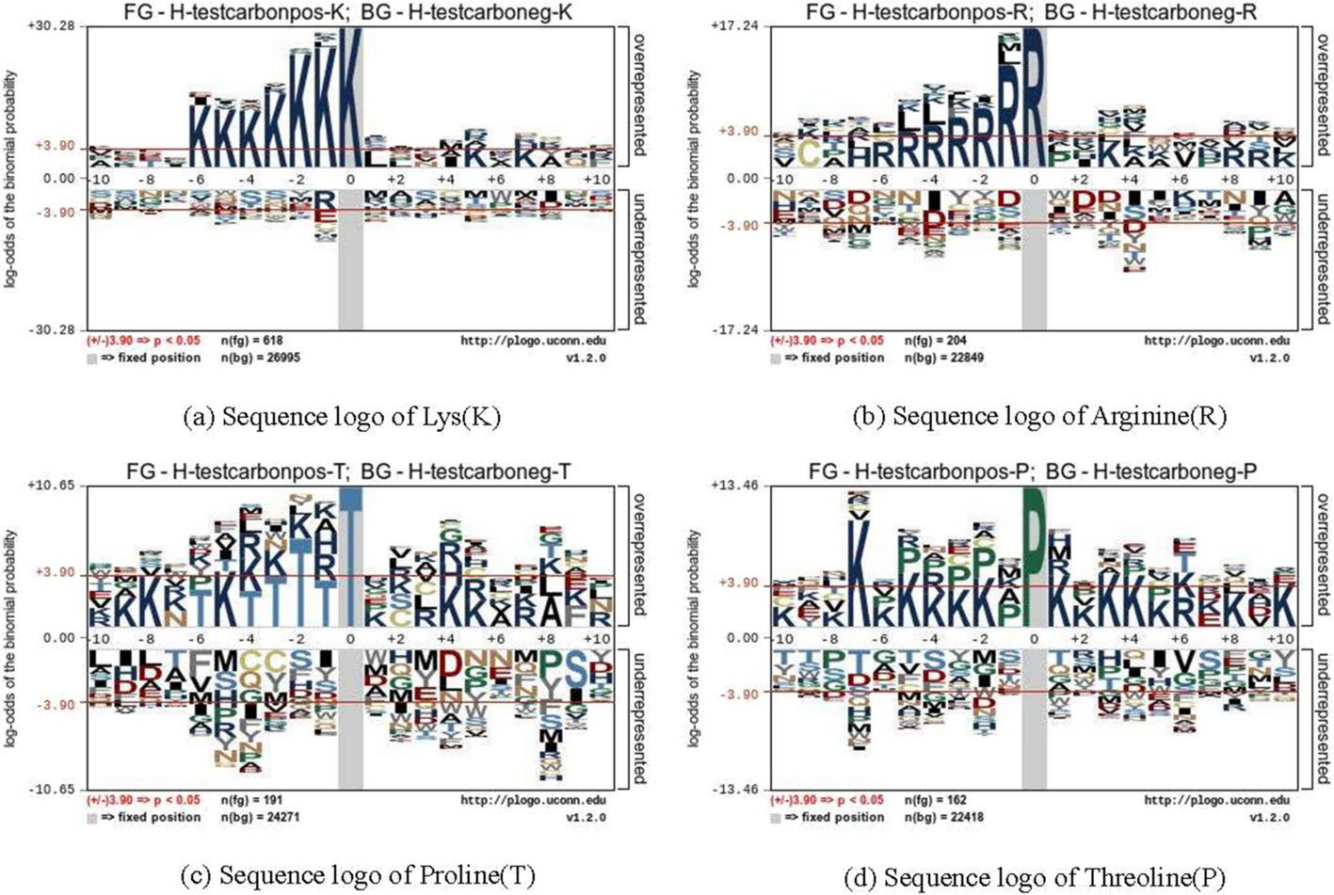
Sequence logo of four residues in test dataset

### Dataset

We collected the human protein carbonylation data from CarSPred as the training dataset. It contains 266 K, 119 R, 116 T, and 114 P carbonylation sites and 1802 K, 754 R, 702 T, and 716 P non-carbonylation sites respectively. At the same time, we also collected the human protein carbonylation data from Carsite2 as the test dataset. It contains 618 K, 204 R, 191 T, 162 P carbonylation sites and 6995 K, 2849 R, 4271 T, 2418 P non-carbonylation sites respectively. The training dataset was originally derived from the carbonylated protein database doi: org/10.1371/journal.pone.0111478.s001 collected and established by Hongqiang Lv et al. in 2014. The test dataset was originally derived from the carbonylated protein database doi: 10.1093/bioinformatics/bty123 collected and established by R.Shyama Prasad Rao et al. in 2018. The two databases are completely different. Then we submitted these datasets to the pLogo web server [26] (https://pLogo.uconn.edu/), and the sequence logo of four residues are shown in Figs. 5 and 6. Table 2 summaries the detailed information of datasets used in experiments.

**Table 2:** The details of datasets

Dataset	Class	Carbonylation type and number of samples			
		K	R	T	P
Training dataset	Positive	266	119	116	114
	Negative	1802	754	702	716
Test dataset	Positive	618	204	191	162
	Negative	26995	22849	24271	22418

### Evaluate performance of model

The performance of model was evaluated using the following three measurements: Sensitivity (Sen), Specificity (Spe), and the area under the receiver operating characteristic curves (AUC), which were defined as follows:

11 $$\begin{aligned}{} & {} Sen\,\,=\,\,1 -\,\,\frac{TP}{P}, \end{aligned}$$

12 $$\begin{aligned}{} & {} Spe\,\,=\,\,1 -\,\,\frac{TN}{N}, \end{aligned}$$

13 $$AUC = \frac{{\sum\nolimits_{{i = 1}}^{P} {\sum\nolimits_{{j = 1}}^{N} {f\left( {P_{{score_{i} }} ,N_{{score_{j} }} } \right)} } }}{{P \times N}},f\left( {x_{i} ,x_{j} } \right) = \left\{ {\begin{array}{*{20}l} {1,{\text{ }}} \hfill & {x_{i} {\text{ }}} \hfill & {gt;x_{j} ;{\text{ }}} \hfill \\ {0,{\text{ }}} \hfill & {{\text{ }}{\text{.}}} \hfill & {otherwise{\text{ }}} \hfill \\ \end{array} } \right.{\text{ }}$$

where *P* represents the number of positive samples, *N* represents the number of negative samples. *TP* indicates the number of positive samples which are predicted as positive samples. *TN* indicates the number of negative samples which are predicted as negative samples. $P_{score_i}$ is the probability score of the i-th sample in positive samples set. $N_{score_j}$ is the probability score of the j-th sample in negative samples set.

### Implementation details

We use ten-fold cross-validation to test the reliability of the classifier’s classification results. We divide the training set into 10 partitions. For each partition *i*, train the model on the remaining 9 partitions and then evaluate the model on partition *i*. The final result of training set is equal to the average of 10 results. Then we set the number of iterations to 200, record and save the optimal model during the model iteration process. Finally, the optimal model is used to obtain the result of test set.

In addition, the software packages used in this paper are as follows:

- Pse-in-One-2.0
- Pycharm-community-2017.3.2
- Python(3.7.0)
- Torch(1.5.0)
- Sklearn(1.0.1)
- Numpy(1.21.5)

### Compare with other resampling methods

**Fig. 7:**
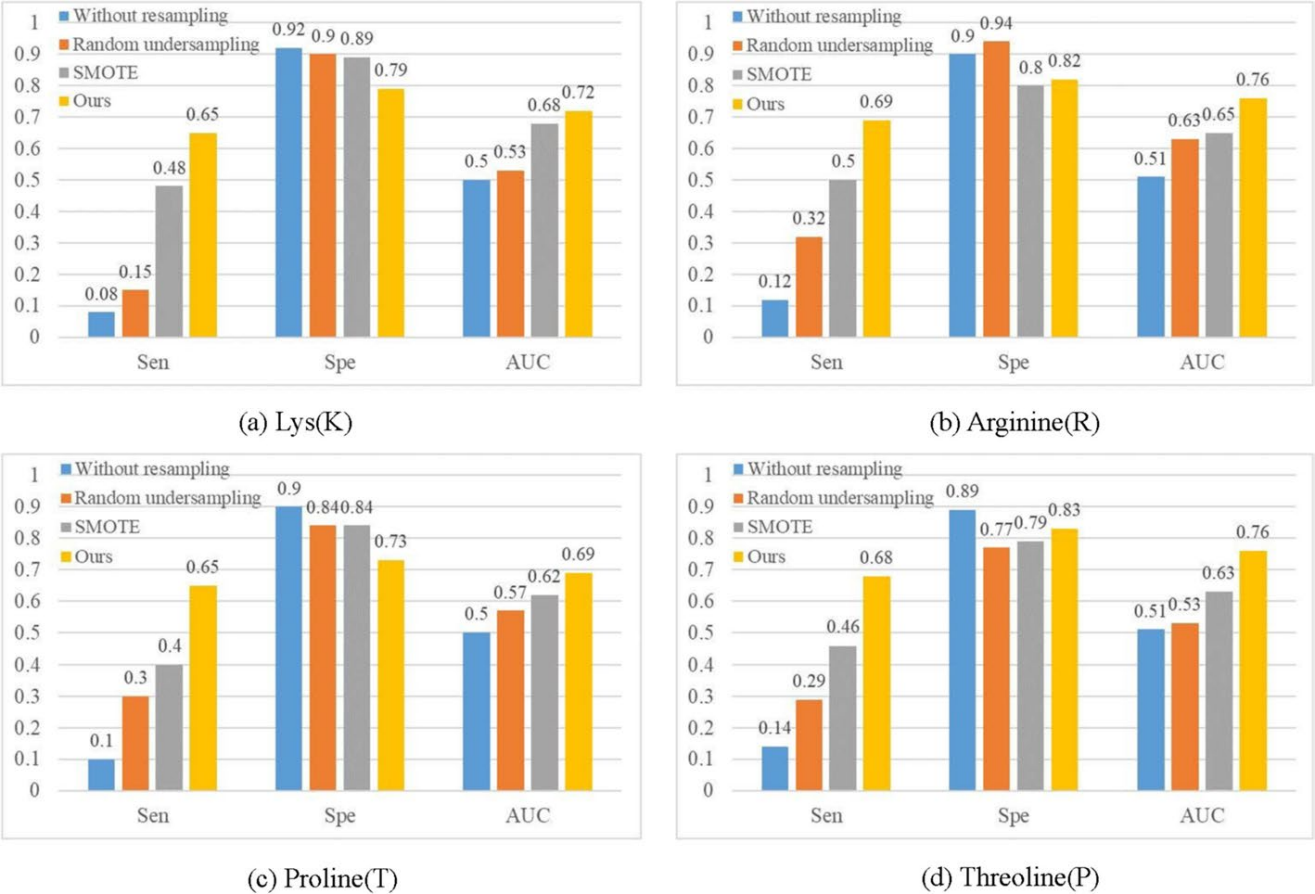
Comparison of different resampling methods for K/R/T/P carbonylation sites

To verify the effectiveness of Carsite_AGan resampling, we compared our approach with three resampling methods including conducting without resampling, SMOTE oversampling and random undersampling for the training dataset. The comparison results are shown in Fig. 7.

From Fig. 7, we can see the values of sensitivity and AUC of our strategy are obviously higher than that of others on the four carbonylation sites, but the specificity value of our strategy is commonly lower than that of others. The dataset of the four carbonylation sites has a very high imbalance rate, which will cause the classification results of others to favor the negative. Experimental results demonstrate that our strategy is more suitable for alleviating the extreme class imbalance carbonylation site data than other strategies.

### Compare with other carbonylation sites prediction methods

To verify the carbonylation prediction effectiveness of our approach, we compared our approach with four kinds of carbonylation prediction methods:

- PTMPred [7]. It employs a support vector machine with the kernel matrix computed by position-specific propensity matrices.
- CarSPred [8]. It employs four types of features and an mRNA feature selection algorithm with a weighted support vector machine.
- CarSPred.Y [10]. It employs three types of features and an IFS feature selection algorithm with a weighted support vector machine.
- CarSite [14]. It employs a one-sided selection undersampling algorithm to balance the training dataset and a hybrid combination of four feature extraction strategies with a support vector machine.

**Fig. 8:**
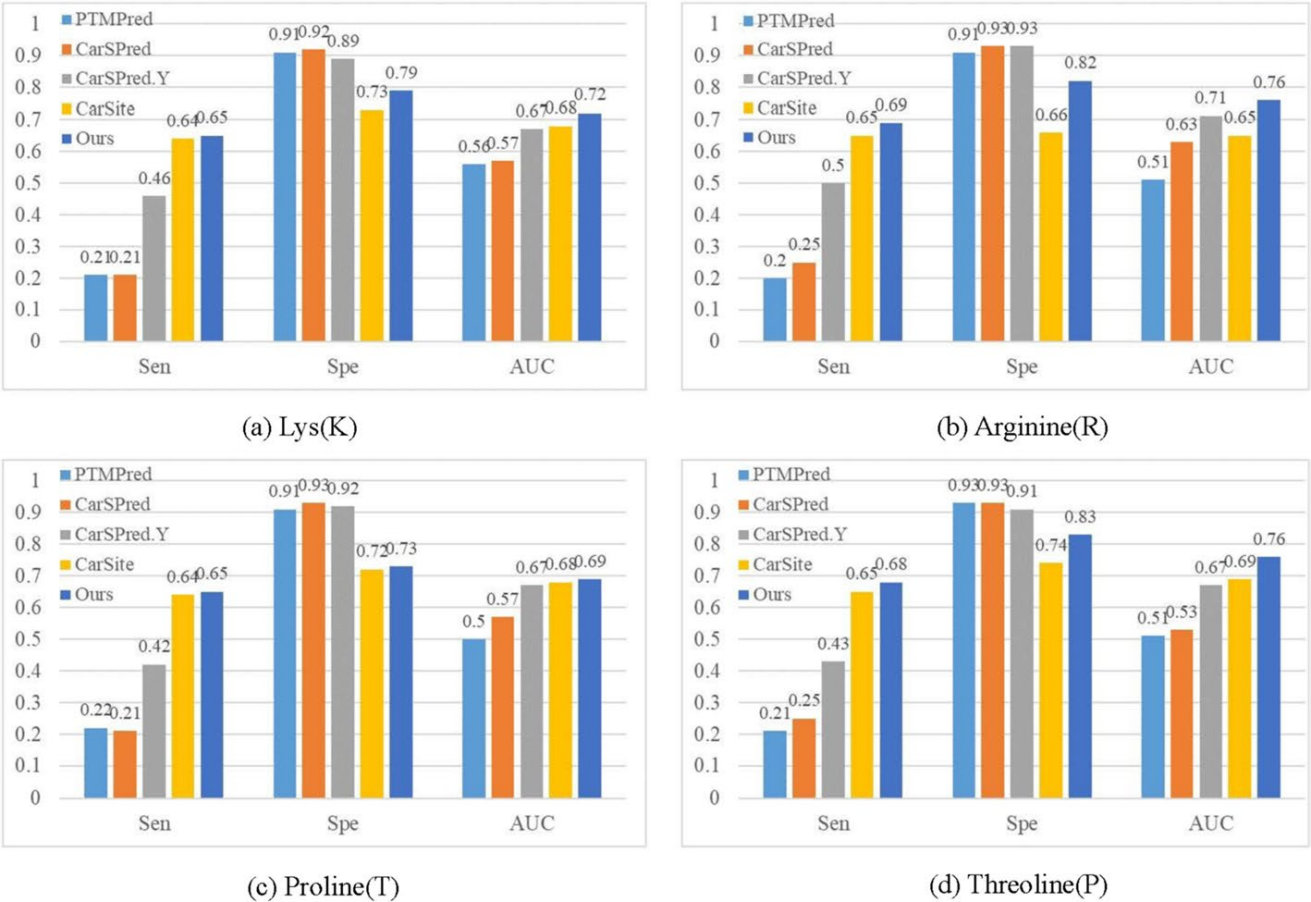
Comparison of different human protein carbonylation prediction methods for K/R/T/P carbonylation sites

The relevant results are shown in Fig. 8. From Fig. 8 we can see that the values of sensitivity and AUC of our strategy are obviously higher than those of PTMPred, CarSPred, and CarSPred.Y on the four carbonylation sites, but the specificity value of our strategy is commonly lower. That’s because the imbalance rate of the four carbonylation site dataset is very high, which will cause the classification results of PTMPred, CarSPred, and CarSPred.Y to favor the negative. In addition, our strategy significantly outperforms CarSite on all evaluation indicators. Experimental results demonstrate that using our approach to rebalanced carbonylation data can significantly improve the effect of identifying protein carbonylation sites.

## Discussion

In this section, to evaluate the effectiveness of the designed modules of our approach, we select 1000 K un-carbonylation sites and 200, 100, 10 and 3 K carbonylation sites from the above training dataset to construct four new imbalanced training datasets whose imbalance rates are respectively 5, 10, 100 and 333. Then we select 500 K un-carbonylation sites and 500 K carbonylation sites from the above test dataset to construct a test dataset. We will discuss how we choose the optimal sampling scale for Carsite_AGan. In addition, we experiment and discuss the effectiveness of the designed modules of our approach, which include a novel attention-based undersampling method and a Gan-based oversampling method.

### Optimal sampling scale for Carsite_AGan

In our Carsite_AGan approach, different sampling scale usually leads to different classification results. How to obtain an optimal sample scale? The state-of-the-art works [27–29] show that: (i) synthesizing numbers of new samples are easy to introduce a large amount of noise data, and (ii) deleting too many original samples will cause serious information loss. In order to balance the numbers of synthesized and deleted samples as much as possible, the sum of oversampling and undersampling ratios should be minimal. Let *s* denote the optimal scale, *s*/*m* denote the ratio of oversampling and *M*/*s* denote the ratio of undersampling. Then *s* needs to satisfy:

14 $$\begin{aligned} Min\left( \frac{s}{m} + \frac{M}{s}\right) , \end{aligned}$$

Here $M> s> m > 0$. Thus, the optimal sampling scale *s* can be calculated as:

15 $$\begin{aligned} s = \sqrt{mM}. \end{aligned}$$

Therefore, we simultaneously sample the number of positive samples and the number of negative samples to the optimal sampling value *s*. The balanced dataset is completely balanced.

**Fig. 9:**
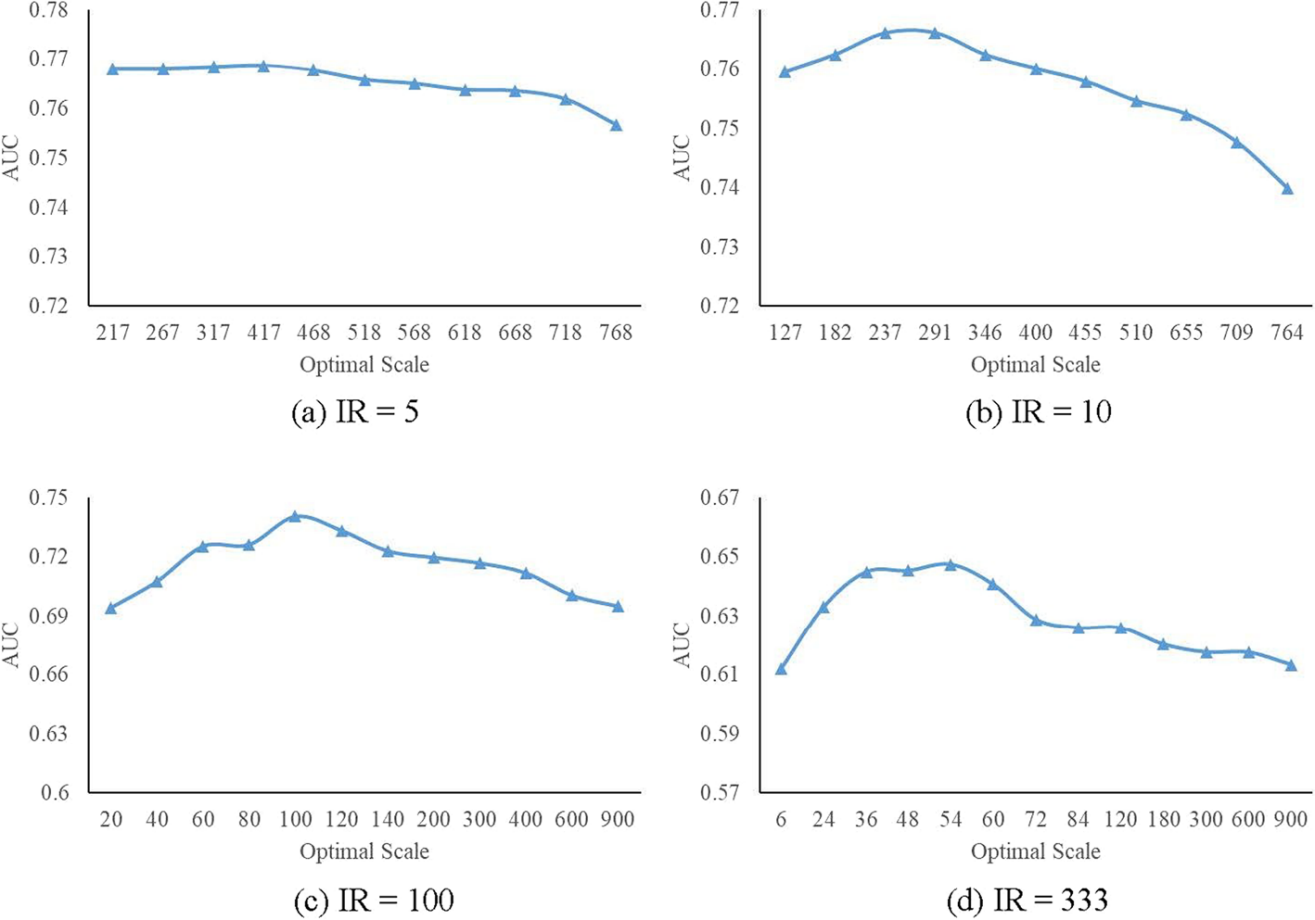
AUC of two-way rebalancing with increasing sampling scales under different IRs. **a**–**d**, IR=5 (**a**), IR=10 (**b**), IR=100 (**c**) and IR=333 (**d**)

Figure 9 shows the AUC classification results of Carsite_AGan with increasing sampling scales under different Imbalance Rate (IR). (a–d), IR=5 (a), IR=10 (b), IR=100 (c) and IR=333 (d). When the optimal sampling scale *s* is taken, the classification results tend to be optimum, where *s*=374 for IR=5, *s*=288 for IR=10, *s*=99 for IR=100 and *s*=55 for IR=333. And the regions around *s* values are generally corresponding to better classification results. Figure 9 verifies the effectiveness of calculating the optimal sampling scale in our approach Carsite_AGan.

### Evaluation of attention-based undersampling

**Fig. 10:**
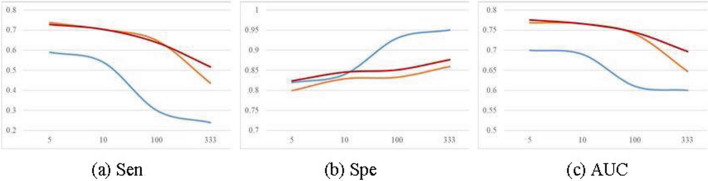
Classification performance of our approach (using Gan), an alternative strategy using random undersampling, and an alternative strategy using SMOTE-based oversampling with increasing imbalance ratio. **a**–**c**, AUC **a**, Spe **b** and Sen **c**, with using random undersampling plus Gan-based oversampling (blue color), using attention-based undersampling plus SMOTE-based oversampling (orange)) and ours that employs attention-based undersampling plus Gan-based oversampling (red)

The attention technique can obtain the value of each sample’s importance. In our approach, we propose the attention-based undersampling to replace the commonly used random undersampling. Figure 10 shows the classification performance of our approach (red color) and an alternative strategy using random undersampling (blue). When IR > 10, the values of Sen and AUC of using random undersampling more obviously drops to 0 than ours, and the value of Spe of using random undersampling more obviously rises to 1. In addition, the values of Sen and AUC of ours are commonly higher than those of using random undersampling. When IR < 10, the value of Spe of ours is slightly higher than that of using random undersampling. However, when IR > 10, the value of Spe of ours is obviously lower than that of using random undersampling. That’s because when the imbalance rate is higher, which will cause the classification results of using random undersampling to favor the negative.

### Evaluation of Gan-based oversampling

Gan is a state-of-the-art sample generating method. In our approach, we employ the Gan-based oversampling to replace the commonly used SMOTE-based oversampling. Figure 10 shows the classification performance of our approach (red color) and an alternative strategy using SMOTE-based oversampling(orange). When IR > 100, our strategy with Gan significantly outperforms the alternative strategy with SMOTE on all evaluation indicators. It demonstrates that a strategy using Gan is more suitable for solving the extreme class imbalance problem than that using SMOTE. When IR $<=$ 100, Spe using Gan outperforms that using SMOTE, while AUC and Sen values using Gan are close to those using SMOTE. The above experimental results show that for the situation of extreme class imbalance, the proposed strategy significantly outperforms commonly-used rebalancing strategies; and for the general class imbalance situations where imbalance ratios are not extremely high, our approach can acquire totally better classification results as compared with commonly-used strategies.

### Evaluation of nonlinear SVM

To compare the impact of different classifiers, we performed ablation experiments. Respectively, we used classification and regression tree (CART), Random Forest (RF), Gradient boosting (GB), Adaptive Boosting (AdaBoost), and Extreme Gradient Boosting (XGBoost) instead of the nonlinear SVM we used to conduct experiments. We used the best result of the training set in 200 interactions as the final result of the training set. The results of training and testing sets of K-carbonylated protein are shown in Table 3.

**Table 3:** The training dataset results and test dataset results after 200 iterations of K carbonylation sites (%)

Method	AUC	Sen	Spe
CART(train)	73.73	66.33	81.13
CART(test)	50.85	50.50	51.20
RF(train)	65.63	58.33	72.93
RF(test)	62.26	55.67	68.85
GB(train)	76.45	69.50	83.40
GB(test)	66.48	59.83	73.13
AdaBoost(train)	78.00	71.00	85.00
AdaBoost(test)	70.65	64.50	77.80
XGBoost(train)	73.36	66.37	80.35
XGBoost(test)	71.35	64.50	78.20
nonlinear SVM(train)	75.48	68.53	82.43
nonlinear SVM(test)	72.83	65.81	79.85

Through experiments, it can be seen that the results of nonlinear SVM are better than other classifiers. This is because CART has an over-fitting problem and requires pre-pruning to solve over-fitting. RF, GB, AdaBoost, and XGBoost use CART as the base classifier. Therefore, their performance is better than CART, but there is still an overfitting problem, and further parameter adjustment is required.

In addition, the Gaussian kernel function we used is also called the radial basis function (RBF). To compare the impact of different types of kernels, we performed a new ablation experiment. Respectively, we used linear kernels and polynomial (poly) kernels instead of the Gaussian kernel used in this paper. We used the best result of the training set in 200 interactions as the final result of the training set. The results of training and testing sets of K-carbonylated protein are shown in Table 4.

**Table 4:** The training dataset results and test dataset results after 200 iterations of K carbonylation sites. (%)

Method	AUC	Sen	Spe
SVM(line)(train)	72.86	65.77	79.95
SVM(line)(test)	67.90	60.00	74.90
SVM(poly)(train)	75.56	68.60	82.52
SVM(poly)(test)	72.55	65.50	79.60
SVM(RBF) (train)	75.48	68.53	82.43
SVM(RBF) (test)	72.83	65.81	79.85

Through experiments, it can be seen that the results of training and testing sets using the Gaussian kernel are better than using the line kernel. This is because using the Gaussian kernel can capture the nonlinear relationship between samples so as to learn more useful information than the line kernel. The results of using the poly kernel on the training set are slightly better than the results of using the Gaussian kernel, but the results of using the poly kernel on the test set are worse than the results of using the Gaussian kernel. This indicates that using the poly kernel is easier overfitting than using Gaussian kernels.

## Conclusion

In this work, we propose a novel protein carbonylation sites prediction approach based on the attention technique and generative adversarial network. Carsite_AGan uses the attention technique to select sites with high importance value from un-carbonylation sites and uses Gan to generate high-feasibility carbonylation sites. The attention technique can obtain the value of each sample’s importance. The Gan can generate high-feasibility samples through its generator and discriminator. Extensive experiments on two human protein carbonylation site datasets and a yeast protein carbonylation site dataset demonstrate that our approach can achieve better performance in identifying carbonylation sites than other competing carbonylation site prediction methods. In addition, experiments also demonstrate the effectiveness of the designed modules of our approach.

We use GAN to generate new samples for positive samples. If the number of positive samples is single digits, then there will be fewer samples for the generator to learn, and the quality of the samples generated will be very poor, leading to serious overfitting problems. This issue deserves research in the future.

## Data Availability

The training dataset is freely available at https://sourceforge.net/projects/hqlstudio/files/CarSPred-1.0/Datasets. The test dataset is freely available at http://47.100.136.41:8081/dataSet. Our strategy code is freely available at https://github.com/CrazyMage313/Carsite_AGan.

## References

[1] Zheng J, Bizzozero O (2010). Traditional reactive carbonyl scavengers do not prevent the carbonylation of brain proteins induced by acute glutathione depletion. Free Radical Res.

[2] Bizzozero OA, DeJesus G, Callahan K, Pastuszyn A (2005). Elevated protein carbonylation in the brain white matter and gray matter of patients with multiple sclerosis. J Neurosci Res.

[3] Muntané G, Dalfó E, Martínez A, Rey M, Avila J, Pérez M, Portero M, Pamplona R, Ayala V, Ferrer I (2006). Glial fibrillary acidic protein is a major target of glycoxidative and lipoxidative damage in pick’s disease. J Neurochem.

[4] Korolainen MA, Auriola S, Nyman TA, Alafuzoff I, Pirttilä T (2005). Proteomic analysis of glial fibrillary acidic protein in Alzheimer’s disease and aging brain. Neurobiol Dis.

[5] Maisonneuve E, Ducret A, Khoueiry P, Lignon S, Longhi S, Talla E, Dukan S (2009). Rules governing selective protein carbonylation. PLoS ONE.

[6] Chen Y, Liu Y, Lan T, Qin W, Zhu Y, Qin K, Gao J, Wang H, Hou X, Chen N (2018). Quantitative profiling of protein carbonylations in ferroptosis by an aniline-derived probe. J Am Chem Soc.

[7] Xu Y, Wang X, Wang Y, Tian Y, Shao X, Wu L-Y, Deng N (2014). Prediction of posttranslational modification sites from amino acid sequences with kernel methods. J Theor Biol.

[8] Lv H, Han J, Liu J, Zheng J, Liu R, Zhong D (2014). Carspred: a computational tool for predicting carbonylation sites of human proteins. PLoS ONE.

[9] Jia J, Liu Z, Xiao X, Liu B, Chou K-C (2016). icar-psecp: identify carbonylation sites in proteins by Monte Carlo sampling and incorporating sequence coupled effects into general pseaac. Oncotarget.

[10] Lv H, Liu J, Han J, Zheng J, Liu R (2016). A computational method to predict carbonylation sites in yeast proteins. Genet Mol Res.

[11] Hasan MAM, Li J, Ahmad S, Molla MKI (2017). predcar-site: carbonylation sites prediction in proteins using support vector machine with resolving data imbalanced issue. Anal Biochem.

[12] Weng S-L, Huang K-Y, Kaunang FJ, Huang C-H, Kao H-J, Chang T-H, Wang H-Y, Lu J-J, Lee T-Y (2017). Investigation and identification of protein carbonylation sites based on position-specific amino acid composition and physicochemical features. BMC Bioinformatics.

[13] Kao H-J, Weng S-L, Huang K-Y, Kaunang FJ, Hsu JB-K, Huang C-H, Lee T-Y (2017). Mdd-carb: a combinatorial model for the identification of protein carbonylation sites with substrate motifs. BMC Syst Biol.

[14] Zuo Y, Jia C-Z (2017). Carsite: identifying carbonylated sites of human proteins based on a one-sided selection resampling method. Mol BioSyst.

[15] Zuo Y, Lin J, Zeng X, Zou Q, Liu X (2021). Carsite-ii: an integrated classification algorithm for identifying carbonylated sites based on k-means similarity-based undersampling and synthetic minority oversampling techniques. BMC Bioinformatics.

[16] Zhou T, Rong J, Liu Y, Gong W, Li C (2022). An ensemble approach to predict binding hotspots in protein-RNA interactions based on SMOTE data balancing and random grouping feature selection strategies. Bioinformatics.

[17] Cateni S, Colla V, Vannucci M (2014). A method for resampling imbalanced datasets in binary classification tasks for real-world problems. Neurocomputing.

[18] Colla V, Matarese N, Reyneri LM. A method to point out anomalous input-output patterns in a database for training neuro-fuzzy system with a supervised learning rule. In: 2009 Ninth international conference on intelligent systems design and applications, 2009;pp. 1307–1311. IEEE.

[19] Creswell A, White T, Dumoulin V, Arulkumaran K, Sengupta B, Bharath AA (2018). Generative adversarial networks: an overview. IEEE Signal Process Mag.

[20] Rastgoo R, Kiani K, Escalera S (2021). Sign language recognition: a deep survey. Expert Syst Appl.

[21] Popel M, Tomkova M, Tomek J, Kaiser Ł, Uszkoreit J, Bojar O, Žabokrtskỳ Z (2020). Transforming machine translation: a deep learning system reaches news translation quality comparable to human professionals. Nat Commun.

[22] Wan Z, Zhang B, Chen D, Zhang P, Wen F, Liao J (2022). Old photo restoration via deep latent space translation. IEEE Trans Pattern Anal Mach Intell.

[23] Deng Y, Zhang T, Lou G, Zheng X, Jin J, Han Q-L (2021). Deep learning-based autonomous driving systems: a survey of attacks and defenses. IEEE Trans Industr Inf.

[24] Sebald DJ, Bucklew JA (2000). Support vector machine techniques for nonlinear equalization. IEEE Trans Signal Process.

[25] Liu B, Xu J, Zou Q, Xu R, Wang X, Chen Q (2014). Using distances between top-n-gram and residue pairs for protein remote homology detection. BMC Bioinform.

[26] O’shea JP, Chou MF, Quader SA, Ryan JK, Church GM, Schwartz D (2013). plogo: a probabilistic approach to visualizing sequence motifs. Nat Methods.

[27] Megahed FM, Chen Y-J, Megahed A, Ong Y, Altman N, Krzywinski M (2021). The class imbalance problem. Nat Methods.

[28] Oksuz K, Cam BC, Kalkan S, Akbas E (2021). Imbalance problems in object detection: a review. IEEE Trans Pattern Anal Mach Intell.

[29] Arefeen MA, Nimi ST, Rahman MS (2022). Neural network-based undersampling techniques. IEEE Trans Syst Man Cybernet Syst.

